# Psoriasis in the Brazilian Public Health System: A Nationwide Analysis of Healthcare Utilization and Inequalities, 2014–2024

**DOI:** 10.3390/healthcare14101338

**Published:** 2026-05-13

**Authors:** Suzieni Padoin Zuccolo De Bortoli, Anber Ancel Tanaka, Rafael Aguilar Magalhães, João Victor Gonçalves Aques, João Lucas Volc, Douglas Andreas Valverde, Valderilio Feijó Azevedo

**Affiliations:** 1 Postgraduate Program in Internal Medicine and Health Sciences, Department of Internal Medicine, Federal University of Paraná (UFPR), Curitiba 80060-000, PR, Brazil; valderilio@hotmail.com; 2 Dermatology Service, Mackenzie Evangelical University Hospital, Curitiba 80730-000, PR, Brazil; anbertanaka@gmail.com; 3 Postgraduate Program in Numerical Methods in Engineering, Sector of Exact Sciences and Technology, Federal University of Paraná (UFPR), Curitiba 81531-980, PR, Brazil; rafael.aguilar@ufpr.br; 4 Department of Internal Medicine, Federal University of Paraná (UFPR), Curitiba 80060-000, PR, Brazil; joaogaques@gmail.com (J.V.G.A.); joao.volc@ufpr.br (J.L.V.); 5 Techtrials, São Paulo 05517-050, SP, Brazil; doug.valverde@ttrials.com

**Keywords:** psoriasis, healthcare utilization, health inequalities, health services accessibility, administrative data, Brazil

## Abstract

**Highlights:**

**What are the main findings?**

- A nationwide analysis of the Department of Informatics of the Brazilian Unified Health System identified 54,950 patients with psoriasis receiving care in the Brazilian unified health system (2014–2024), with predominance of psoriasis vulgaris and female patients.
- Marked regional disparities and temporal variations were observed, including a reduction in healthcare encounters in 2020 followed by recovery through 2024.

**What are the implications of the main findings?**

- Administrative healthcare data can be used to monitor disease burden and healthcare utilization in large, universal health systems.
- Regional differences highlight the need for targeted public health strategies to improve access and equity in psoriasis care.

**Abstract:**

**Background:** Psoriasis (PsO) is a chronic inflammatory disease associated with substantial healthcare needs. In Brazil, nationwide evidence based on administrative data remains limited, particularly regarding healthcare utilization and inequalities in the public health system. **Objectives:** The objective was to analyze nationwide patterns of healthcare utilization and to explore potential regional and sociodemographic inequalities in access to care among patients with PsO within the Brazilian Unified Health System (SUS) from 2014 to 2024. **Methods:** This retrospective observational study used nationwide administrative data from DATASUS, the health information system of the Brazilian Unified Health System. Patients with at least one healthcare encounter coded with ICD-10 L40 between 2014 and 2024 were included. Sociodemographic characteristics, clinical subtypes, geographic distribution, and temporal trends in healthcare utilization were analyzed. Registered healthcare prevalence for 2024 was estimated. Results: A total of 54,950 unique patients were identified. Psoriasis vulgaris was the most frequent subtype (38.8%). Women accounted for 57.7% of cases, and the most represented age group was 46–60 years. Most patients were classified as White (52.7%) or mixed race (35.1%). Higher case concentrations were observed in the Southeast and Northeast, with higher healthcare prevalence in the Southeast and South. Healthcare encounters declined in 2020, followed by recovery through 2024, with regional variation in registered healthcare prevalence, highlighting persistent inequalities. **Conclusions:** This nationwide real-world study highlights patterns of healthcare utilization and regional inequalities in psoriasis care within Brazil’s public health system. Administrative data provide valuable insights to support health policy and resource allocation in universal healthcare settings.

## 1. Introduction

Psoriasis (PsO) is a chronic, immune-mediated, systemic inflammatory disease classified among noncommunicable diseases. It is characterized by a relapsing course and a substantial impact on quality of life [1,2]. Clinically, PsO presents with different phenotypes, of which plaque psoriasis is the most common, accounting for approximately 80–90% of cases [1]. Beyond cutaneous involvement, PsO is associated with relevant comorbidities, including metabolic syndrome and cardiovascular disease, further reinforcing its relevance as a public health concern [2,3,4].

The global burden of PsO has been widely documented, with an increasing number of cases and a considerable impact on years lived with disability, partly driven by demographic changes and improved access to diagnosis [5,6,7]. However, the burden of PsO is determined not only by disease occurrence but also by inequalities in access to diagnosis, specialist care, and treatment. These disparities are particularly relevant in low- and middle-income countries, where healthcare system organization and social determinants strongly influence patterns of healthcare utilization and diagnostic recognition [8,9,10].

In Brazil, substantial inequalities persist in the distribution of healthcare resources, specialist workforce, infrastructure, and access to services [11,12,13,14], reflecting broader structural differences within the Brazilian Unified Health System (Sistema Único de Saúde, SUS) [15,16]. Such disparities may result in delayed diagnosis, undertreatment, and limited access to advanced therapies for chronic inflammatory diseases such as psoriasis, disproportionately affecting socially and geographically vulnerable populations [9,10].

Evidence on PsO in Brazil remains limited and methodologically heterogeneous. Previous Brazilian studies have included a geographic survey based on telephone interviews conducted in Brazilian capitals and regional analyses based on outpatient administrative records, providing important but distinct perspectives on psoriasis burden and healthcare access [17,18]. However, nationwide longitudinal analyses integrating administrative records at the individual patient level and specifically exploring regional inequalities within SUS remain scarce. To our knowledge, no previous nationwide study using data from the Department of Informatics of the Brazilian Unified Health System (DATASUS) has comprehensively evaluated PsO through integrated individual-level outpatient and hospital records. Unlike previous regional analyses or studies based on isolated outpatient records, the present study uses nationwide administrative data from both settings, allowing identification of unique patients and a broader evaluation of healthcare utilization patterns and regional inequalities within SUS over time.

Administrative health databases are valuable for understanding healthcare utilization and disease distribution within health systems; however, they primarily capture patients who access care and receive a coded diagnosis [19,20,21,22,23]. Therefore, such data should be interpreted as reflecting registered healthcare prevalence rather than true population prevalence. This distinction is particularly relevant in settings with unequal access to specialist care, where lower observed prevalence may reflect barriers to diagnosis and healthcare utilization rather than lower disease occurrence [18].

Accordingly, this study aimed to analyze patterns of healthcare utilization and to explore potential inequalities in access to care among patients with PsO within SUS during 2014–2024, using nationwide administrative health data.

## 2. Methods

This nationwide, retrospective, observational descriptive study with a quantitative approach used publicly available secondary administrative data from the Brazilian Unified Health System (SUS). We analyzed healthcare records from 1 January 2014 to 31 December 2024, extracted in March 2025, when the databases were considered complete for the study period, using the Techtrials Disease Explorer^®^ platform developed by Techtrials^TM^ (Techtrials Healthcare Data Science, [São Paulo] Brazil) [24].

Data extraction and processing were performed using the Techtrials Disease Explorer^®^ platform, which enables automated ingestion, standardization, and consolidation of large-scale administrative data from the Department of Informatics of the Brazilian Unified Health System (DATASUS), including the Outpatient Information System (SIA/SUS) and the Hospital Information System (SIH/SUS) [24,25]. The platform performs automated duplicate detection, record-format validation, cross-variable consistency checks, and longitudinal integrity checks to ensure stable linkage of encrypted patient identifiers over time and across systems. No proprietary clinical definitions or diagnostic algorithms were used for case identification. All analytic definitions were based exclusively on variables publicly available in official DATASUS databases, and the final statistical analyses were conducted using reproducible Python scripts developed independently by the research team, allowing full conceptual reproducibility using public DATASUS data without dependence on proprietary classification rules. As an internal validation step, consistency between patient counts, temporal distributions, and regional aggregation across SIA/SUS and SIH/SUS was systematically assessed during data harmonization. Additional quality-control procedures included verification of duplicate linkage stability, compatibility between demographic variables and encounter history, and manual inspection of extreme values identified during exploratory analyses.

All data were anonymized at the source and contained no personally identifiable information, in accordance with the Brazilian General Data Protection Law (Law No. 13,709/2018) [26]. Each individual was represented in DATASUS by a unique encrypted numeric identifier that was consistent across SIA/SUS and SIH/SUS and stable over time. Unique patients were defined as distinct encrypted identifiers. For patient-level analyses, multiple records corresponding to the same identifier (including repeated visits and records present in both systems) were consolidated into a single individual, whereas healthcare utilization analyses were conducted at the encounter (record) level. Records with missing or invalid identifiers were excluded from analyses requiring individual-level linkage.

Patients were included if they had at least one healthcare encounter recorded in SIA/SUS or SIH/SUS during the study period with a diagnosis of psoriasis identified using the International Classification of Diseases, 10th Revision (ICD-10), category L40 or any L40 subcode [27]. The use of at least one recorded diagnosis was intentionally adopted to maximize sensitivity for capturing healthcare utilization patterns within a universal public health system, acknowledging the inherent limitations of administrative data for diagnostic confirmation. This approach prioritizes healthcare-system capture rather than strict clinical case confirmation and is consistent with the study objective of describing healthcare utilization and access inequalities rather than estimating true population prevalence.

Because single-record definitions may increase the risk of diagnostic misclassification due to coding errors, rule-out diagnoses, or transient administrative registration, this choice was interpreted cautiously and supported by prior validation studies showing that ICD-based case definitions in administrative databases involve a trade-off between sensitivity and specificity [20,21,22,23]. In our dataset, 51.7% of patients had exactly one psoriasis-related healthcare record and 67.0% had up to two records during the entire study period, with a median of 1 record (interquartile range: 1–4), indicating that stricter case definitions could substantially exclude patients with intermittent but clinically plausible healthcare utilization. As an exploratory sensitivity analysis, a stricter definition requiring at least two psoriasis-related healthcare encounters was evaluated. Under this criterion, 27,287 patients remained eligible, corresponding to a 50.3% reduction compared with the primary study population (54,950 patients), supporting the decision to retain the ≥1-record criterion for the main analysis.

The following ICD-10 subcodes were considered: L40.0 (plaque psoriasis), L40.1 (generalized pustular psoriasis), L40.2 (acrodermatitis continua), L40.3 (palmoplantar pustulosis), L40.4 (guttate psoriasis), L40.8 (other psoriasis), and L40.9 (psoriasis, unspecified), as well as the aggregated L40 code when recorded without specification of a subcategory, which is a frequent occurrence in administrative databases. The selection of diagnostic codes was based on the World Health Organization classification and the Brazilian Ministry of Health Clinical Protocol and Therapeutic Guidelines for Psoriasis [28].

**Figure 1:**
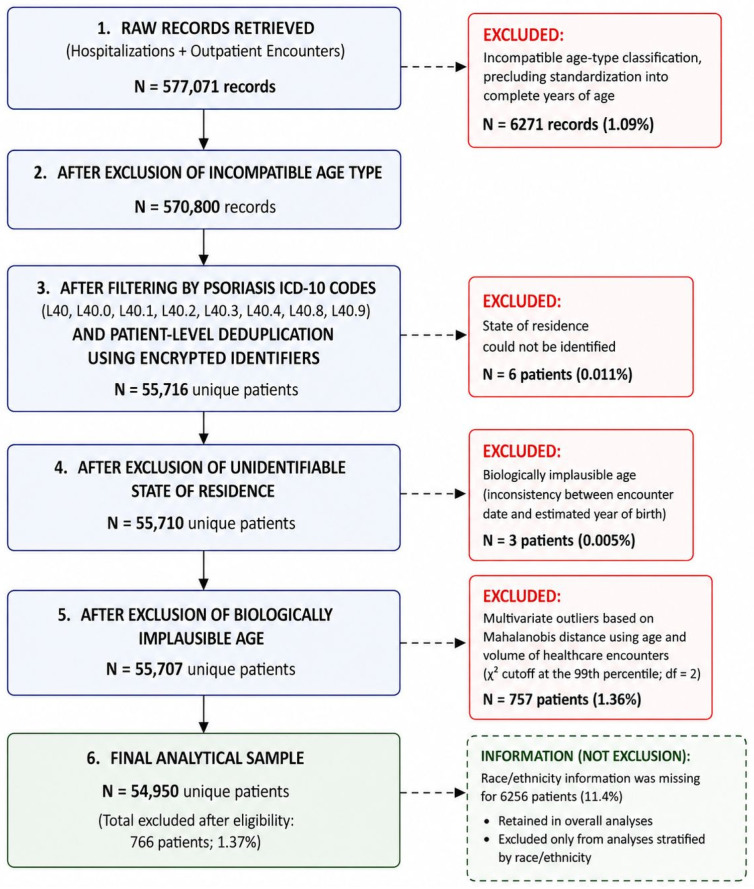
Flowchart of data processing and construction of the final analytical sample of patients with psoriasis in the Brazilian Unified Health System (SUS), 2014–2024. Note: Percentages were calculated in relation to the immediately preceding step. Patient deduplication was performed using encrypted individual identifiers. ICD-10 codes included L40, L40.0, L40.1, L40.2, L40.3, L40.4, L40.8, and L40.9.

From a total of 577,071 inpatient and outpatient records initially retrieved, 6271 records were excluded because they presented an incompatible age-type classification, precluding standardization into complete years of age. After filtering for psoriasis-related ICD-10 codes (L40, L40.0, L40.1, L40.2, L40.3, L40.4, L40.8, and L40.9) and patient-level deduplication using encrypted identifiers, 55,716 unique patients remained eligible. Subsequently, 6 patients were excluded because the state of residence could not be identified (0.011%), and 3 patients were excluded due to biologically implausible age resulting from inconsistencies between encounter date and estimated year of birth (0.005%). In addition, 757 patients (1.36%) were excluded after multivariate outlier detection using Mahalanobis distance based on age and volume of healthcare encounters, applying a χ^2^ cutoff at the 99th percentile (df = 2). This approach was preferred over univariate Z-score methods to preserve patients with clinically plausible intensive healthcare utilization, such as those with greater disease severity or complex care needs. Patient identifiers were previously validated by the Techtrials platform, and no records with missing or invalid identifiers were identified at this stage. The final analytical sample comprised 54,950 unique patients (total excluded after eligibility: 766; 1.37%). Race/ethnicity information was missing for 6256 patients (11.4% of the final analytical sample); these patients were retained in overall analyses but excluded from analyses stratified by race/ethnicity. Figure 1 summarizes the sequential data-cleaning procedures, eligibility criteria, and exclusions applied to construct the final analytical sample.

For temporal analyses, the index date was defined as the earliest recorded encounter with a psoriasis-related code (L40) for each individual during the study period.

Variables extracted from administrative records included sex, age group, race/ethnicity, and state of residence. Patients were assigned to Brazilian regions and states according to the recorded state of residence. Registered healthcare prevalence was estimated for each Brazilian state for 2024 as the number of unique patients with at least one psoriasis-related healthcare encounter recorded in SUS systems in 2024, divided by the estimated resident population of the same state and year according to the Brazilian Institute of Geography and Statistics (IBGE), and expressed per 100,000 inhabitants [29]. This measure reflects recorded healthcare utilization and diagnostic capture within administrative systems and should not be interpreted as true population prevalence of PsO. Throughout the manuscript, the term registered healthcare prevalence refers exclusively to this healthcare-system-based measure.

Statistical analyses were conducted using reproducible scripts in Python (version 3.12.2) [30]. Categorical variables were summarized as counts and percentages and were compared using the chi-square test, with Monte Carlo methods applied when appropriate. Continuous variables were analyzed using the Mann–Whitney U test. Because the primary objective was descriptive epidemiological characterization of healthcare utilization patterns, multivariable modeling was not included in the main analysis. Regional differences were therefore interpreted descriptively as indicators of healthcare access and system organization rather than as causal determinants of disease occurrence. All tests were two-sided, and statistical significance was defined as *p* < 0.05.

Ethics approval and informed consent were waived because this study exclusively used publicly available, anonymized secondary administrative data, with no possibility of individual identification. The study was conducted in accordance with Brazilian National Health Council Resolution No. 510/2016 and the Brazilian General Data Protection Law (Law No. 13,709/2018) [31].

## 3. Results

**Figure 2:**
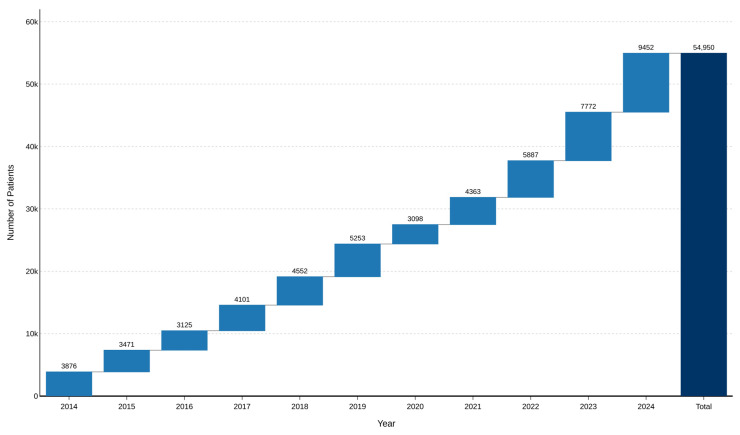
Annual number of patients with psoriasis in SUS, Brazil, 2014–2024. Note: The figure shows the annual number of unique patients with psoriasis who had a first recorded psoriasis-related healthcare encounter in SUS healthcare information systems during 2014–2024, as well as the cumulative total over the study period. These values reflect healthcare utilization and diagnostic capture within the public health system and should not be interpreted as true disease incidence or true population prevalence of psoriasis.

During 2014–2024, a total of 54,950 unique patients diagnosed with PsO were identified within the SUS. The annual number of unique patients with a first recorded psoriasis-related healthcare encounter increased over time, from 3876 in 2014 to 9452 in 2024, with a marked disruption in 2020 followed by progressive recovery in subsequent years (Figure 2).

The largest annual increments were observed in 2022, 2023, and 2024 (5887; 7772; and 9452 new patients, respectively), reflecting progressive expansion in healthcare-system capture and diagnostic registration rather than necessarily a true increase in disease occurrence at the population level.

In 2020, the number of patients with a first recorded psoriasis-related healthcare encounter decreased from 5253 in 2019 to 3098, corresponding to a 41.0% reduction compared with the previous year. Compared with the mean annual number observed during the pre-pandemic period of 2017–2019 (4635 patients/year), this represented a reduction of 33.2%. To evaluate deviation from the expected temporal trend, a Poisson regression model was fitted using data from 2014 to 2019, showing an expected annual growth of 8.15% (IRR = 1.082; *p* < 0.001). Based on this model, the projected number of patients for 2020 was 5298 (95% CI: 5156–5444). The observed value (3098) was entirely below the expected confidence interval, corresponding to a 41.5% deficit relative to the projected trend.

**Figure 3:**
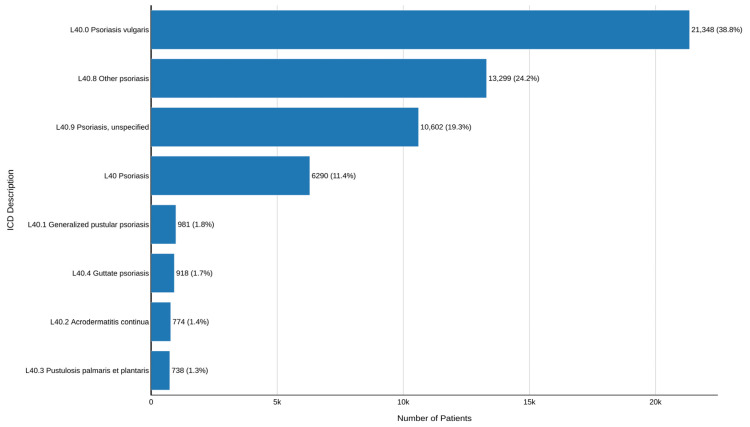
Distribution of psoriasis patients by ICD-10 codes in SUS, Brazil, 2014–2024. Note: The figure illustrates the distribution of unique patients with psoriasis according to ICD-10 diagnostic codes within the SUS during 2014–2024. Data are presented as absolute numbers and percentages for each diagnostic category, including specific and non-specific codes.

Regarding diagnostic distribution, psoriasis vulgaris (L40.0) was the most frequent diagnosis (21,348; 38.8%), followed by L40.8 (other psoriasis) (13,299; 24.2%) and L40.9 (psoriasis, unspecified) (10,602; 19.3%). The aggregated code L40 was identified in 6290 patients (11.4%). Less frequent clinical forms included L40.1, L40.4, L40.2, and L40.3, each accounting for less than 2% of patients (Figure 3).

**Figure 4:**
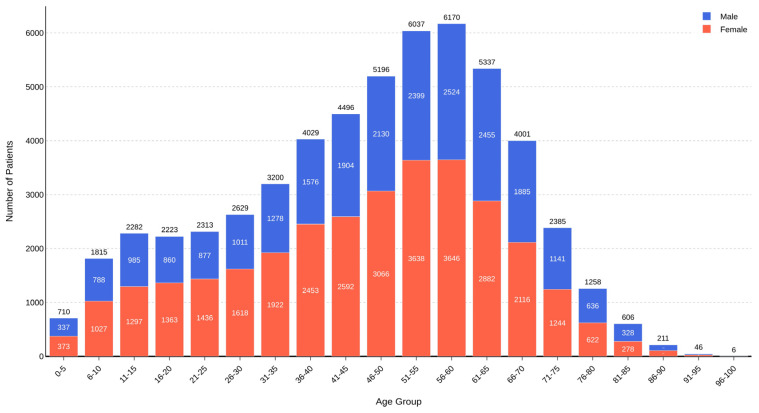
Distribution of psoriasis patients by sex and age in SUS, 2014–2024. Note: The figure shows the distribution of unique patients with psoriasis stratified by sex and age groups within the SUS during 2014–2024, expressed as absolute numbers of patients.

In demographic analyses, 31,714 patients (57.7%) were female and 23,236 (42.3%) were male. Mean age was 45.7 years (median: 49) among women and 47.2 years (median: 50) among men, with a statistically significant difference in age distribution between sexes (Mann–Whitney U test; *p* < 0.0001). The most frequent age groups were 56–60 years (6170; 11.2%), 51–55 years (6037; 11.0%), and 46–50 years (5196; 9.5%). When broader age categories were analyzed, 30,998 patients (56.4%) were aged 25–59 years, 15,088 (27.5%) were aged ≥60 years, and 8864 (16.1%) were aged <25 years. A statistically significant association between sex and age group was observed (χ^2^ = 222.51; *p* < 0.0001) (Figure 4).

Among the 48,694 patients with available race/ethnicity information, 25,686 (52.7%) were classified as White, 17,077 (35.1%) as Brown (mixed race), 3134 (6.4%) as Asian, 2765 (5.7%) as Black, and 32 (0.07%) as Indigenous. No statistically significant association was observed between sex and race/ethnicity distribution (χ^2^ = 3.657; *p* = 0.4544). Race/ethnicity information was missing for 6256 patients (11.4% of the final analytical sample). Missingness did not differ significantly by sex (female: 11.2%; male: 11.6%; χ^2^ = 2.33; *p* = 0.127). By geographic region, significant heterogeneity was observed (χ^2^ = 1036.7; *p* < 0.001), with the highest proportion of missing data in the Central-West region (25.5%) and the lowest in the North (7.6%) and South (6.3%). Across age groups, variation was statistically significant but clinically modest, ranging from 10.1% among older adults to 12.8% among children (χ^2^ = 40.6; *p* < 0.001).

**Figure 5:**
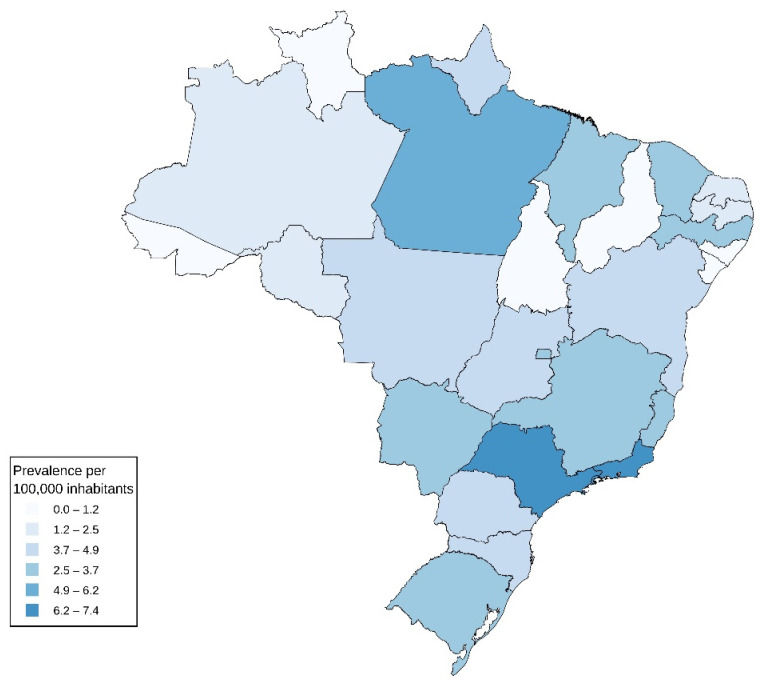
Registered healthcare prevalence of psoriasis by state in SUS, Brazil, 2024. Note: The choropleth map displays the registered healthcare prevalence of psoriasis per 100,000 inhabitants within the SUS across Brazilian states in 2024. This measure reflects the proportion of unique patients with a recorded psoriasis diagnosis in SUS healthcare systems relative to the estimated population of each state, based on official population estimates.

In geographic analyses, the largest proportion of patients was observed in the Southeast region (32,155; 58.5%), followed by the Northeast (8130; 14.8%), South (7606; 13.8%), Central-West (3823; 7.0%), and North (3236; 5.9%). At the state level, São Paulo accounted for 23,427 patients (42.6%), followed by Rio de Janeiro (4246; 7.7%), Paraná (3835; 7.0%), and Minas Gerais (3771; 6.9%). The lowest proportions were observed in Acre (12; 0.02%), Roraima (15; 0.03%), and Amapá (40; 0.07%). When registered healthcare prevalence per 100,000 inhabitants was considered, higher values were observed in the Southeast and South regions (Figure 5).

## 4. Discussion

This nationwide administrative analysis provides a comprehensive description of the epidemiology of PsO within a large public healthcare system. In line with recent global estimates, the absolute number of individuals living with PsO has increased substantially over recent decades, rising from approximately 23 million in 1990 to more than 43 million in 2021. This growth has been primarily attributed to population ageing and expanded access to diagnosis, whereas age-standardized prevalence rates have remained relatively stable over time [6,7,32]. In this context, our findings suggest that the expansion observed in the SUS time series predominantly reflects increased healthcare demand, improved diagnostic capacity, and greater visibility of PsO within health systems, rather than a proportional increase in the underlying population occurrence of the disease [6,7,33]. Importantly, these findings reinforce that administrative data in universal health systems primarily capture patterns of healthcare utilization and do not directly reflect the true underlying population distribution of disease [19]. This distinction is critical for interpreting registered healthcare prevalence and supports its use in informing policy, optimizing resource allocation and identifying inequities in chronic disease management.

By characterizing PsO using healthcare system records from the SUS, this study helps address gaps in the national epidemiological literature, which has historically relied on self-reported population surveys or regional analyses with limited scope [17,18]. Our findings complement existing population-based estimates and highlight the value of integrating healthcare system-based and population-based approaches as distinct yet complementary strategies to better characterize disease burden, particularly in middle-income settings where epidemiological data remain limited [8,34].

The temporal analysis showed a progressive increase in the number of unique patients up to 2019, followed by an abrupt decline in 2020 and subsequent recovery. This pattern is consistent with the impact of the COVID-19 pandemic on healthcare utilization, as international studies reported marked reductions in dermatology consultations and newly recorded psoriasis cases during periods of sanitary restrictions [35,36,37]. Accordingly, the disruption observed in 2020 likely reflects temporary barriers to healthcare access rather than a true change in disease occurrence. The observed number of patients in 2020 was 41.5% lower than expected based on the pre-pandemic trend and remained below projected levels in 2021, supporting the interpretation of a statistically significant disruption in healthcare utilization during the pandemic period. The subsequent increase observed from 2022 onward, with values exceeding projected levels in 2023 and 2024, suggests gradual restoration of healthcare access combined with possible recovery of previously delayed diagnoses and expansion of diagnostic capture within the SUS.

The predominance of psoriasis vulgaris observed in this study is consistent with international literature, which indicates that this phenotype accounts for approximately 80–90% of cases, whereas less frequent variants such as pustular, guttate, and palmoplantar psoriasis remain relatively rare [1,2]. The high frequency of non-specific diagnostic codes in our dataset further underscores known limitations of administrative databases and suggests underuse of more detailed diagnostic classifications, a phenomenon widely described in validation studies [20,21,22,32]. Despite this limitation, the overall distribution of clinical subtypes remains aligned with established epidemiological patterns.

The predominance of female patients slightly diverges from population-based studies, in which PsO prevalence is generally similar between sexes, but is consistent with healthcare utilization patterns [23,38]. Women are known to access outpatient and specialized care more frequently, including dermatology services, in both high-income settings and Brazil [39,40,41]. Therefore, the observed sex distribution may partially reflect differential healthcare-seeking behavior and greater use of outpatient and specialist services by women, rather than necessarily true differences in disease occurrence.

The age distribution, with a higher concentration among middle-aged adults, is consistent with the known epidemiological profile of PsO, which is characterized by a bimodal onset pattern and increasing prevalence with age [6,7,34]. This pattern is consistent with the chronic and cumulative nature of PsO and with increased healthcare utilization over time among individuals with longer disease duration [42,43,44].

Differences observed by race/ethnicity should be interpreted with caution. The predominance of White individuals and the substantial proportion of missing data may reflect both inequalities in access to healthcare and limitations in data recording. International evidence indicates underdiagnosis and reduced access to dermatological services among non-White populations, which are associated with structural barriers and gaps in clinical training for diverse skin phototypes [45,46,47,48]. In addition, inconsistencies in administrative data recording may compromise estimate accuracy and contribute to underestimation of the healthcare burden in these groups [49]. Accordingly, race/ethnicity in this context should be interpreted primarily as a marker of healthcare access and data capture rather than as a direct proxy for the true population distribution of PsO in Brazil.

From a geographic perspective, the findings show marked regional differences in registered healthcare prevalence, with higher concentrations of patients and higher registered healthcare prevalence in the Southeast and South regions. National studies indicate that these regions have greater healthcare infrastructure, higher workforce density, and improved access to specialized services, whereas other regions face more substantial access barriers [12,14,50]. National workforce data from *Medical Demographics in Brazil 2025* show that 56.9% of dermatologists in Brazil are concentrated in the Southeast region, whereas only 14.0% are located in the Northeast and 3.7% in the North, with marked differences in specialist density per 100,000 inhabitants across states [51]. In addition, 60.3% of dermatologists are in state capitals, reinforcing the concentration of specialized care in urban centers and the barriers faced by patients living in smaller municipalities and underserved regions. The unequal distribution of healthcare resources and specialized services likely contributes to delayed diagnosis, underdiagnosis, and lower administrative capture of psoriasis in these areas [13,51,52,53]. These findings suggest that reducing regional inequalities in psoriasis care within SUS may depend not only on expanding specialist availability but also on strengthening referral pathways in primary care, improving access to teledermatology support, and optimizing regional organization of dermatology services. Policies aimed at decentralizing specialist care and improving diagnostic capacity outside major urban centers may help reduce underdiagnosis and improve earlier therapeutic access [13,14,15,51,52,53].

These findings are consistent with international literature showing that geographic variation in studies based on healthcare system data largely reflects differences in health-system organization, resource availability, and diagnostic capture, rather than true biological variation in disease occurrence [5,7,32,34]. Similarly, European studies based on national administrative databases conducted in France and Germany have shown that such systems enable characterization of demographic and clinical patterns of PsO, primarily reflecting healthcare organization and diagnostic practices rather than intrinsic epidemiological differences [54,55]. Taken together, these observations support the interpretation of administrative data as a tool for assessing healthcare system performance and access, rather than as a direct measure of disease occurrence.

From a public health perspective, these results should be interpreted within a framework of health inequities in access to care. While inequalities refer to observable differences between population groups, inequities represent systematic, avoidable, and unjust differences driven by social, economic, and structural determinants [56,57]. In this context, the disparities identified in this study reflect not only differences in healthcare utilization but also potential inequities in access to specialized care within the SUS. In practical terms, this means that lower registered healthcare prevalence in underserved regions should not be interpreted as lower disease burden but potentially as evidence of diagnostic delay, reduced access to specialist evaluation, and lower administrative capture within the health system. Administrative epidemiology in this context becomes a marker of health-system performance, as much as of disease registration [19,56,57].

The healthcare burden of PsO, combined with its chronic nature and frequent association with systemic comorbidities, reinforces its relevance as a public health concern [3,4,58,59,60]. These findings highlight the need for strategies to expand access to diagnosis, improve the quality of clinical and administrative data, and reduce regional disparities. Strengthening specialist distribution, integrating teledermatology initiatives, improving referral pathways between primary and specialized care, and promoting more consistent diagnostic coding may represent feasible opportunities within SUS to improve equity in psoriasis care and optimize resource allocation [13,14,15,51,52,53].

This study has limitations inherent to the use of administrative data, including dependence on healthcare utilization patterns, variability in diagnostic coding, and the absence of detailed clinical information such as disease severity and patient-reported outcomes. In addition, the findings reflect the registered healthcare prevalence of PsO within the SUS and therefore do not allow direct estimation of population prevalence. Accordingly, lower observed frequencies in specific regions or subgroups should not be interpreted as evidence of lower disease occurrence, but may instead reflect barriers to diagnosis, reduced healthcare utilization, and limitations in administrative registration. Regional comparisons should also be interpreted cautiously, as this descriptive analysis did not adjust for differences in age structure, urbanization, dermatologist availability, healthcare infrastructure, private healthcare coverage, or other contextual factors across Brazilian states. These factors may influence diagnostic capture and healthcare utilization patterns and may partially confound geographic comparisons. Severity bias should also be considered, as patients with more active or extensive disease are more likely to be captured in healthcare system records, whereas milder cases may be under-represented [20,21,22,32]. Furthermore, it was not possible to distinguish incident from prevalent cases or to determine the interval between symptom onset and the first recorded healthcare encounter, which limited more precise temporal inferences.

Despite these limitations, the nationwide coverage of the SUS, its standardized structure, and the large volume of available data confer robustness to the analyses, allowing the identification of structural patterns and inequalities at scale that are difficult to capture in clinical cohorts or regional studies. These results provide evidence to inform health policy and dermatologic care planning in Brazil, reinforcing the need to promote equity in access to services, improve health information systems, and strengthen surveillance and care strategies for PsO within the SUS [15]. The predominance of non-specific diagnostic codes further highlights opportunities to improve coding practices and healthcare surveillance, with potential implications for resource allocation and the development of more effective public health policies.

## 5. Conclusions

This nationwide analysis of PsO within the SUS identified 54,950 unique patients between 2014 and 2024, with predominance of psoriasis vulgaris, female patients, and middle-aged adults, as well as marked regional differences in patient distribution and registered healthcare prevalence. Higher concentrations of recorded cases and higher healthcare prevalence were observed in the Southeast and South regions, highlighting important geographic disparities in access to diagnosis and specialized care.

These findings indicate that the observed distribution of PsO in administrative databases is strongly influenced by healthcare utilization patterns, diagnostic capacity, and health-system organization, rather than directly representing true population prevalence. From a public health perspective, strengthening health information systems, improving the quality of clinical coding, and expanding equitable access to dermatologic care across regions are essential steps to reduce inequalities and support better resource allocation within universal healthcare systems such as the SUS.

Future studies should explore more specific case definitions and longitudinal approaches to better distinguish incident from prevalent cases, as well as integrate clinical outcomes, treatment patterns, and comorbidity burden. Further investigation of regional barriers to dermatologic care may also help clarify how healthcare system organization influences the observed inequalities in psoriasis management.

## Data Availability

The data that support the findings of this study are publicly available from the Brazilian Unified Health System information platform (DATASUS), maintained by the Brazilian Ministry of Health (http://datasus.saude.gov.br). The data were accessed and processed through the Techtrials Disease Explorer^®^ platform. The datasets are fully anonymized and contain no direct personal identifiers; therefore, individual-level identification is not possible.

## Abbreviations

The following abbreviations are used in this manuscript:

**Abbreviation**	**Definition**
ANS	National Supplementary Health Agency (Agência Nacional de Saúde Suplementar)
COVID-19	Coronavirus disease 2019
DATASUS	Department of Informatics of the Brazilian Unified Health System (Departamento de Informática do Sistema Único de Saúde)
GBD	Global Burden of Disease
IBGE	Brazilian Institute of Geography and Statistics (Instituto Brasileiro de Geografia e Estatística)
ICD-10	International Classification of Diseases, 10th Revision
LGPD	Brazilian General Data Protection Law (Lei Geral de Proteção de Dados Pessoais)
PCDT	Clinical Protocol and Therapeutic Guidelines (Protocolo Clínico e Diretrizes Terapêuticas)
PsO	psoriasis
SIA/SUS	Outpatient Information System of the Unified Health System (Sistema de Informações Ambulatoriais do SUS)
SIH/SUS	Hospital Information System of the Unified Health System (Sistema de Informações Hospitalares do SUS)
SUS	Brazilian Unified Health System (Sistema Único de Saúde)
WHO	World Health Organization
